# Shared genetic loci connect cardiovascular disease with blood pressure and lipid traits in East Asian populations

**DOI:** 10.3389/fgene.2025.1635378

**Published:** 2025-06-24

**Authors:** Peng Zhong, Chumeng Zhang, Qinfeng Wu, Xiao Chang

**Affiliations:** ^1^ Department of Cardiology, Jining No.1 People’s Hospital, Jining, Shandong, China; ^2^ The Second School of Clinical Medicine of Shandong First Medical University, Tai’an, Shandong, China; ^3^ College of Medical Information and Artificial Intelligence, Shandong First Medical University, Jinan, Shandong, China; ^4^ The Department of Otorhinolaryngology Head and Neck Surgery, Yantai Yuhuangding Hospital, Qingdao University, Qingdao, Shandong, China

**Keywords:** heart failure, cardiovascular diseases, multi-trait GWAS, cardiometabolic traits, genetic correlation

## Abstract

**Introduction:**

Cardiovascular diseases (CVDs), including myocardial infarction (MI), heart failure (HF), atrial fibrillation (AF), and arrhythmia, are major contributors to global mortality and often share overlapping risk factors and pathophysiological mechanisms. While genome-wide association studies (GWAS) have identified many loci for individual CVDs, the shared genetic architecture across related traits—particularly in East Asian populations—remains underexplored.

**Materials and methods:**

We integrated large-scale GWAS summary statistics from East Asian populations to perform genome-wide and local genetic correlation analyses across four CVD phenotypes and five cardiometabolic traits (blood pressure and lipid levels). Using stratified LD score regression, we assessed tissue-specific heritability enrichment. Multi-trait analysis of GWAS (MTAG) was then employed to identify pleiotropic loci associated with multiple traits, with functional annotation and expression quantitative trait loci (eQTL) data used to explore biological relevance.

**Results:**

We observed extensive genetic correlations among CVDs and between CVDs and cardiometabolic traits, with HF showing the strongest connections to both MI and arrhythmia. Notable genome-wide correlations were found between MI and SBP (rg = 0.35, *P* = 1.59 × 10^−14^) and between HF and DBP (rg = 0.54, *P* = 9.84 × 10^−9^). Stratified heritability analyses revealed significant enrichment in heart and arterial tissues, highlighting the relevance of cardiovascular-specific regulatory elements. MTAG identified several pleiotropic loci, including established genes such as *APOB* and *MC4R*, and novel East Asian-enriched signals such as *QSOX2* and *GUCY1A1*/*GUCY1B1*. Functional data indicated that *QSOX2* variants regulate gene expression in arterial and cardiac tissues, implicating redox regulation in HF and hypertension pathogenesis.

**Conclusion:**

Our findings provide comprehensive insight into the shared genetic determinants of cardiovascular and metabolic diseases in East Asian populations. The identification of pleiotropic and ancestry-specific loci, along with tissue-specific regulatory patterns, underscores the need for integrative multi-trait and population-informed approaches in cardiovascular genetics and risk prediction.

## 1 Introduction

Cardiovascular diseases (CVDs), including myocardial infarction (MI), heart failure (HF), atrial fibrillation (AF), and arrhythmia, represent leading causes of morbidity and mortality worldwide (Kim, 2021). These conditions share overlapping pathophysiological mechanisms, such as myocardial ischemia, structural remodeling, and electrophysiological instability, which are increasingly understood to have genetic underpinnings. Genome-wide association studies (GWAS) have successfully identified susceptibility loci for individual conditions, especially in populations of European ancestry (Hartiala et al., 2021). However, the transferability of these findings to other populations, particularly East Asians, remains limited.

Given the substantial global burden and frequent co-occurrence of MI, HF, AF, and other arrhythmias, increasing attention has been directed toward their shared genetic architecture. Epidemiological data demonstrate considerable comorbidity across these conditions. For instance, up to 30% of patients with HF also develop AF, and prior MI significantly increases the risk of both arrhythmia and progressive HF (Packer, 2020; Wang et al., 2023). Furthermore, AF is now recognized as both a consequence and a driver of structural heart disease, with overlapping risk factors such as hypertension, diabetes, and aging (Li et al., 2022). These observations suggest that these CVDs may not be entirely distinct but instead represent manifestations of interconnected biological processes with common genetic underpinnings.

To systematically investigate these connections, multi-trait analytical frameworks offer a powerful approach to uncover shared and distinct loci contributing to this spectrum of disorders. In particular, Multi-Trait Analysis of GWAS (MTAG) enables the joint interrogation of genetically correlated phenotypes, thereby enhancing locus discovery and improving interpretability of pleiotropic associations (Turley et al., 2018).

In this study, we performed a multi-trait GWAS focusing on four major cardiovascular conditions—MI, HF, AF, and arrhythmia—in East Asian populations. By integrating functional annotations and tissue-specific enrichment analyses, we aimed to elucidate the shared and unique genetic determinants of these disorders and provide ancestry-informed insights into their biological mechanisms.

## 2 Materials and methods

### 2.1 GWAS data

GWAS summary statistics for the traits analyzed in this study were obtained from the GWAS Catalog (Buniello et al., 2019), with detailed information provided in Supplementary Table S1. Summary data for cardiometabolic traits—including systolic blood pressure (SBP), diastolic blood pressure (DBP), high-density lipoprotein (HDL), low-density lipoprotein (LDL), and triglycerides (TG)—were derived from a meta-analysis of East Asian cohorts (Chen et al., 2023), incorporating 92,615 participants from the Taiwan Biobank, Biobank Japan, and the East Asian subset of UK Biobank.

### 2.2 Global genetic correlation analysis

We assessed the genetic correlation (rg) between CVDS and cardiometabolic traits using linkage disequilibrium score regression (LDSC). LDSC estimates rg by analyzing the relationship between GWAS test statistics and LD scores, which represent the cumulative LD between a given SNP and its neighboring variants. This method operates on summary-level GWAS data, making it well-suited for large-scale meta-analyses and robust to confounding from sample overlap. In contrast to approaches requiring individual-level genotypes, LDSC is less susceptible to biases from population stratification or cryptic relatedness. As such, it offers a reliable framework for characterizing the genetic overlap between traits and for informing the selection of phenotypes in subsequent causal inference analyses (Bulik-Sullivan et al., 2015). The formula used in LDSC is as follows:

$$E\left[\beta_{j}\gamma_{j}\right]=\frac{\sqrt{N_{1}N_{2}}r_{g}}{M}l+\frac{N_{s}r}{\sqrt{N_{1}N_{2}}}$$

where $\beta_{j}$ and $\gamma_{j\;}$ represent the effect sizes of ${SNP}_{j}$ on the two traits being tested, $N_{1}$ and $N_{2}$ are the sample sizes for the two traits, $N_{s}$ is the number of overlapping samples between the two traits, r is the phenotypic correlation in the overlapping samples, and, $l_{j}$ is the LD score. In this analysis, precomputed LDSC for HapMap3 SNPs, derived from individuals of European ancestry in the 1000 Genomes Project (Bulik-Sullivan et al., 2015), were used. Variants with an imputation INFO score greater than 0.9 were included for the analysis (Bulik-Sullivan et al., 2015).

### 2.3 Cell-type-specific enrichment of SNP heritability

Stratified LD Score Regression (s-LDSC) extends the LDSC framework by quantifying the contribution of specific genomic annotations to the heritability of complex traits (Trynka et al., 2013). By modeling heritability across predefined functional categories while adjusting for linkage disequilibrium, s-LDSC enables the identification of biologically relevant regions contributing to disease risk. Unlike conventional approaches that rely on genome-wide significant variants, s-LDSC utilizes all SNPs to provide a more comprehensive view of the polygenic architecture. This method is computationally scalable and suitable for large-scale GWAS datasets. Importantly, s-LDSC can pinpoint enrichment in cell type–specific regulatory elements and functional annotations, aiding in the prioritization of genomic features for follow-up studies. Its integration into cross-trait analyses also allows for more nuanced interpretation of genetic correlations by revealing functional categories that drive shared heritability (Finucane et al., 2015).

To dissect tissue- and cell type-specific contributions to trait heritability, we employed s-LDSC using functional annotations derived from six chromatin marks (DHS, H3K27ac, H3K36me3, H3K4me1, H3K4me3, and H3K9ac) across 88 tissues and cell types from the Roadmap Epigenomics Consortium (Finucane et al., 2015). For each histone mark, annotations were grouped into nine biological categories: adipose, cardiovascular, central nervous system, digestive, immune/blood, liver, pancreas, musculoskeletal/connective tissue, and others. Trait-specific heritability enrichment was calculated for each annotation, and the results were visualized through hierarchical clustering based on normalized enrichment scores. This approach enabled the discovery of distinct enrichment profiles across traits and tissues, revealing shared biological underpinnings among genetically correlated phenotypes. Chromatin mark-specific signals provided further granularity, highlighting regulatory elements that may play central roles in tissue-relevant pathways. Together, these analyses support the functional interpretation of GWAS findings and inform the prioritization of candidate tissues and regulatory mechanisms involved in complex trait etiology.

### 2.4 Local genetic correlation analysis

To complement the genome-wide genetic correlation estimates obtained from LDSC, we applied ρ-HESS to assess local genetic correlations between trait pairs (Shi et al., 2017). While LDSC provides a genome-wide average estimate, ρ-HESS partitions the genetic covariance across 1,703 approximately independent genomic regions, enabling locus-level interrogation of shared genetic architecture. This method uses GWAS summary statistics and accounts for linkage disequilibrium patterns and potential sample overlap, without assuming specific distributions of effect sizes. For each region, ρ-HESS estimates local SNP heritability and covariance by projecting GWAS effect size vectors onto LD-derived eigenvectors. We applied this approach to all trait pairs with significant global genetic correlations from LDSC, using a Bonferroni-corrected threshold (*P* < 0.05/1,703) to determine statistical significance. This analysis allowed us to identify specific genomic intervals that disproportionately contribute to the observed genome-wide correlations, revealing heterogeneity in shared genetic architecture across loci.

### 2.5 Multi-trait analysis of GWAS

To enhance locus discovery and statistical power, we applied Multi-Trait Analysis of GWAS (MTAG), a method that leverages shared genetic architecture across genetically correlated traits to identify trait-specific SNP associations (Turley et al., 2018). MTAG uses GWAS summary statistics and estimates pairwise genetic correlations via LDSC (Bulik-Sullivan et al., 2015), accounting for sample overlap and trait correlation to generate unbiased SNP-level effect estimates. In our analysis, the included GWAS datasets were largely derived from non-overlapping cohorts; nonetheless, MTAG’s internal framework corrects for any residual overlap using covariance estimates from LDSC, thereby reducing inflation in test statistics. This framework is particularly suited for complex, polygenic traits with overlapping etiology. After stringent variant filtering, we retained SNPs that achieved genome-wide significance (*P* < 5 × 10^−8^) in MTAG and showed suggestive associations (*P* < 0.01) in the original single-trait GWAS, ensuring robustness and biological relevance. This approach enabled the identification of pleiotropic loci contributing to multiple cardiovascular phenotypes.

## 3 Results

### 3.1 Genetic correlations between cardiovascular diseases and cardiometabolic traits

**FIGURE 1:**
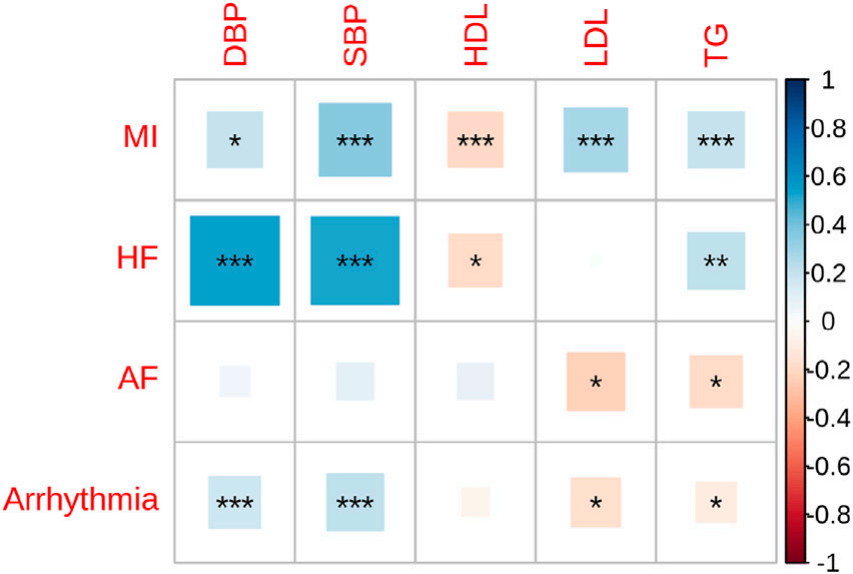
Genetic correlations between cardiovascular diseases and cardiometabolic traits in East Asians. This heatmap illustrates the pairwise genetic correlation between four cardiovascular disease phenotypes—myocardial infarction (MI), heart failure (HF), atrial fibrillation (AF), and arrhythmia—and six cardiometabolic traits—diastolic blood pressure (DBP), systolic blood pressure (SBP), high-density lipoprotein cholesterol (HDL), low-density lipoprotein cholesterol (LDL), triglycerides (TG), and HDL. The strength and direction of correlations are color-coded, with positive correlations shown in blue and negative correlations in red. The size of the squares represents the magnitude of the genetic correlation. Asterisks indicate levels of statistical significance: *P* < 0.05 (*), *P* < 0.01 (**), *P* < 0.001 (***).

We performed genome-wide genetic correlation analysis using LDSC to evaluate shared genetic architecture among four major CVDs and five cardiometabolic traits (Figure 1; Supplementary Table S2). MI exhibited strong and significant positive genetic correlations with SBP (rg = 0.35, SE = 0.05, *P* = 1.59 × 10^−14^), DBP (rg = 0.30, SE = 0.05, *P* = 7.02 × 10^−9^), LDL (rg = 0.27, SE = 0.05, *P* = 1.15 × 10^−7^), and TG (rg = 0.21, SE = 0.03, *P* = 1.92 × 10^−10^). A significant inverse correlation was observed with HDL (rg = −0.20, SE = 0.05, *P* = 8.50 × 10^−5^). These results confirm the known involvement of blood pressure and lipid metabolism pathways in MI pathophysiology. HF also showed robust genetic correlations with both SBP (rg = 0.52, SE = 0.0891, *P* = 4.81 × 10^−9^) and DBP (rg = 0.54, SE = 0.0937, *P* = 9.84 × 10^−9^), highlighting a shared genetic basis linked to hemodynamic stress. Additionally, HF was modestly associated with TG (rg = 0.22, SE = 0.0699, *P* = 0.0021), and negatively correlated with HDL (rg = −0.19, SE = 0.0757, *P* = 0.0142), further suggesting convergence on metabolic dysregulation. In contrast, AF demonstrated weaker genetic correlations with cardiometabolic traits, with no significant associations observed for SBP or DBP. Modest but nominal associations were observed with LDL (rg = −0.22, SE = 0.10, *P* = 0.0296) and TG (rg = −0.18, SE = 0.07, *P* = 0.0109), suggesting a partially distinct genetic basis. Arrhythmia showed moderate genetic correlations with SBP (rg = 0.22, SE = 0.0486, *P* = 8.14 × 10^−6^) and DBP (rg = 0.18, SE = 0.0498, *P* = 0.0004), but weaker or no associations with lipid traits, including non-significant correlations with HDL and LDL. A small negative correlation with TG was noted (rg = −0.10, SE = 0.0442, *P* = 0.0185).

### 3.2 Genetic correlations among cardiovascular diseases

To further examine shared genetic architecture across cardiovascular phenotypes, we estimated pairwise genetic correlations among MI, HF, AF, and arrhythmia (Table 1). MI showed a strong genetic correlation with HF (rg = 0.43, SE = 0.10, *P* = 1.76 × 10^−5^), and a moderate correlation with AF (rg = 0.27, SE = 0.11, *P* = 0.0165). However, the correlation between MI and arrhythmia was not significant (rg = 0.07, SE = 0.07, *P* = 0.2953). In contrast, HF exhibited significant genetic overlap with both AF (rg = 0.48, SE = 0.14, *P* = 0.0005) and arrhythmia (rg = 0.55, SE = 0.087, *P* = 3.78 × 10^−10^), indicating common underlying etiological pathways likely related to electrical and structural remodeling. As expected, AF and arrhythmia were highly correlated (rg = 1.0, SE = 0.08, *P* = 1.73 × 10^−43^), reflecting substantial phenotypic and mechanistic overlap.

**TABLE 1:** Genetic correlations among cardiovascular diseases.

Trait 1	Trait 2	*r*_*g*_	SE	*P*
Myocardial infarction	Atrial fibrillation	0.27	0.11	0.017
	Heart failure	0.43	0.10	1.76 × 10^−5^
	Arrhythmia	0.07	0.07	0.3
Arrhythmia	Atrial fibrillation	1	0.08	1.73 × 10^−43^
	Heart failure	0.55	0.09	3.78 × 10^−10^
Atrial fibrillation	Heart failure	0.48	0.14	5 × 10^−4^

r_*g*_, Genetic correlation estimated using LDSC; SE, standard error of the genetic correlation estimate; P, *P*-value indicating the statistical significance of the correlation.

### 3.3 Partitioning heritability and chromatin marker enrichment in cardiovascular diseases and cardiometabolic traits

s-LDSC analysis demonstrated significant heritability enrichment for cardiovascular and cardiometabolic traits in cardiac and vascular tissues, particularly the left ventricle, right atrium, aorta, coronary artery, and tibial artery (Supplementary Figure S1). Among these, MI and SBP exhibited the highest enrichment in coronary and aortic tissues, respectively, consistent with their biological roles. Genomic regions bearing active chromatin marks—such as H3K27ac and H3K4me3—in these tissues were preferentially enriched, indicating that regulatory elements, including enhancers and promoters active in cardiomyocytes and endothelial cells, are likely to harbor causal variants. Notably, the enrichment profiles were broadly consistent across MI and related cardiometabolic traits, pointing to shared regulatory architecture and suggesting that pleiotropic effects may be mediated by tissue-specific regulatory programs. These results underscore the importance of cardiovascular tissues in shaping the genetic basis of MI and its metabolic risk factors, and emphasize the value of investigating the functional roles of these tissue-specific regulatory elements.

### 3.4 Local genetic correlations between cardiovascular diseases and cardiometabolic traits

To further explore the genetic interplay between cardiovascular diseases and cardiometabolic traits, we conducted a regional analysis of local genetic correlations. This analysis revealed several loci with statistically significant genetic overlap between CVD phenotypes and metabolic risk traits. For instance, a positive local genetic correlation was observed between MI and HDL cholesterol on chromosome 12 (Supplementary Table S3), a region enriched with genes implicated in lipid metabolism, including *HNF1A* (Ai-Ghalayini et al., 2020), which is known to affect HDL levels and coronary artery disease risk. In contrast, we identified negative local correlations between MI and LDL cholesterol at multiple loci. On chromosome 9 (Supplementary Table S3), the region includes *ABO*, a gene involved in coagulation and lipid regulation (Li et al., 2015), and SURF4, which facilitates the secretion of lipoproteins such as VLDL and LDL (Wang et al., 2021). Two additional regions on chromosome 19 were noteworthy: the first (positions 9,383,877–11,849,449; Supplementary Table S3) encompasses LDLR, a gene central to LDL clearance and cardiovascular risk (Franceschini et al., 2009); the second (positions 43,862,455–45,579,043; Supplementary Table S3) contains the *APOE* gene cluster (*APOE*, *APOC1*, *APOC2*, and *APOC4*), all of which play essential roles in lipid transport and homeostasis. Notably, genetic variants in *APOE* have been consistently associated with LDL levels and predisposition to cardiovascular disease (Chaudhary et al., 2012).

### 3.5 Multi-trait GWAS analysis identifies pleiotropic loci for cardiovascular diseases and cardiometabolic traits

To identify pleiotropic loci contributing to CVDs and related cardiometabolic traits, we conducted a multi-trait GWAS using MTAG across MI, HF, SBP, DBP, and other relevant traits. This analysis revealed several new genome-wide significant loci (*P* < 5 × 10^−8^) exhibiting consistent, albeit modest, associations across traits (Table 2). Additionally, we identified several previously unreported East Asian-specific loci characterized by substantially higher minor allele frequencies in East Asian populations compared to other populations (Supplementary Table S4). Notably, the locus tagged by rs13414987 (*APOB*) was jointly associated with HF and MI (*P* = 1.35 × 10^−8^), implicating a gene central to lipid metabolism and atherosclerosis (Supplementary Figure S2). Similarly, rs1942867 (*MC4R*) showed pleiotropic effects on HF and MI (*P* = 4.24 × 10^−8^), consistent with *MC4R*’s known role in energy homeostasis and obesity, both critical contributors to cardiac risk (Supplementary Figure S2). Of particular interest, rs3824359 in *QSOX2* was significantly associated with HF and SBP (*P* = 7.06 × 10^−10^), a novel pleiotropic locus not previously reported in cardiometabolic GWAS (Supplementary Figure S2). To explore the functional relevance of rs3824359, we examined eQTL data from GTEx and found that this variant significantly influences *QSOX2* expression across multiple tissues. The lead allele was associated with increased *QSOX2* expression in cultured fibroblasts (*P* = 2.00 × 10^−20^), testis, tibial artery (*P* = 1.60 × 10^−12^), and subcutaneous adipose tissue, as well as in cardiovascular-relevant tissues including the heart (left ventricle and atrial appendage), skeletal muscle, and arterial vasculature (Supplementary Table S5). Notably, the variant also modulated gene expression in esophageal and brain tissues, suggesting a broad regulatory footprint. Given *QSOX2*’s role in oxidative protein folding and redox homeostasis, its upregulation in vascular and cardiac tissues could implicate reactive oxygen species modulation as a shared pathway in HF and blood pressure regulation. These findings nominate *QSOX2* as a previously unrecognized contributor to cardiovascular pathophysiology and highlight the potential of pleiotropic loci to reveal convergent molecular mechanisms underlying complex traits.

**TABLE 2:** New pleiotropic loci identified in multi-trait GWAS of heart failure.

SNP	CHR	BP	A1	A2	pval1	beta1	pval2	beta2	mtag_beta	mtag_pval	Gene	Trait 1	Trait 2
rs13414987	2	20942072	C	A	9.14E-05	−0.08	1.43E-06	0.1	−0.02	1.35E-08	*APOB*	Heart failure	Myocardial infarction
rs1942867	18	60069038	G	A	1.26E-05	0.08	9.04E-05	−0.07	0.02	4.24E-08	*MC4R*		
rs1077534	3	14836067	A	G	3.69E-04	−0.05	7.41E-07	0.02	−0.01	1.25E-08	*FGD5*		Diastolic blood pressure
rs16864620	2	6039940	G	A	5.00E-05	−0.06	4.17E-06	0.02	−0.01	6.90E-09	*SOX11*		Systolic blood pressure
rs3824359	9	136213383	T	C	1.17E-05	0.07	1.41E-06	−0.03	0.01	7.06E-10	*QSOX2*		

SNP: Reference SNP cluster ID from dbSNP database; CHR, Chromosome location (hg38 assembly); BP, Base pair position (hg38 coordinates); A1, Effect allele (tested in association models); A2, Reference allele; pval1, Single-trait GWAS p-value for Trait 1; beta1, Effect size of A1 allele on Trait 1; pval2, Single-trait GWAS p-value for Trait 2; beta2, Effect size of A1 allele on Trait 2; mtag_beta, Multi-trait meta-analysis effect estimate; mtag_pval, Multi-trait analysis p-value; gene, Nearest protein-coding gene.

**FIGURE 2:**
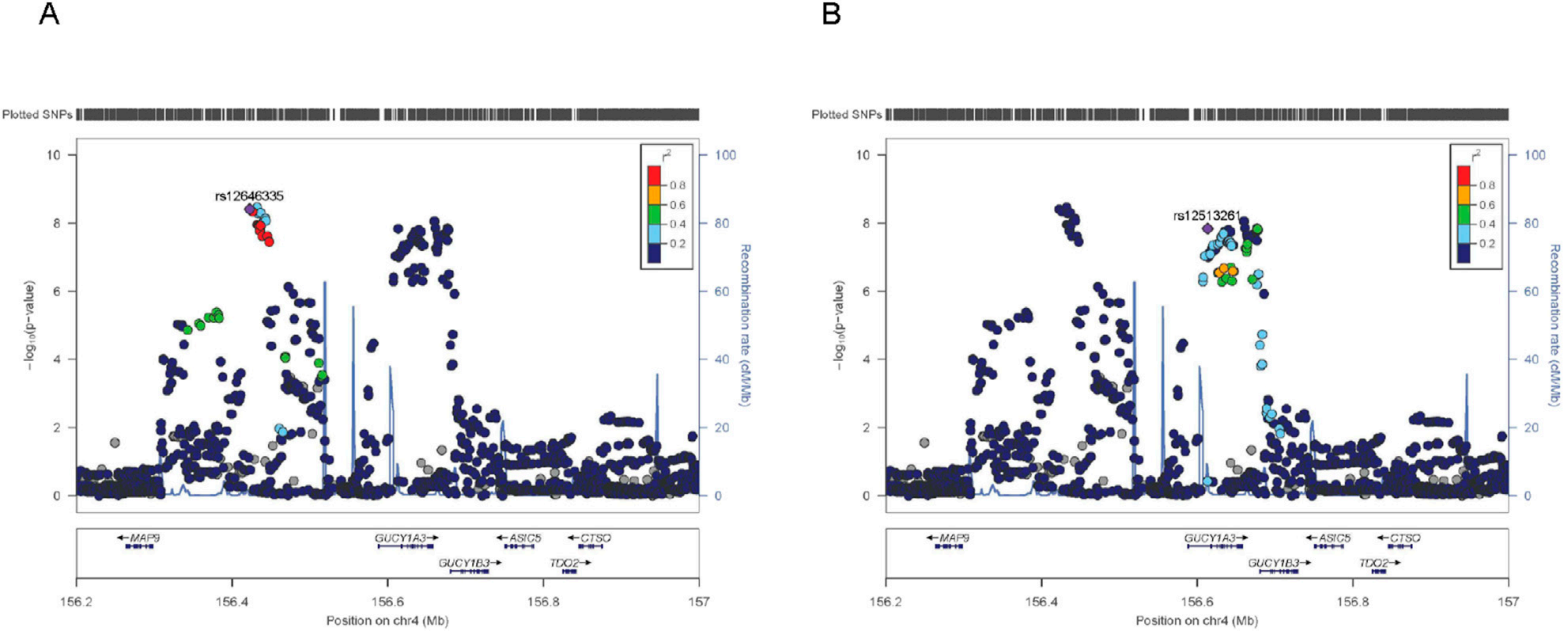
Regional association plots at the GUCY1A1/GUCY1B1 locus. **(A)** Association plot centered on signal 1, using the lead SNP rs12646335 as the index variant. **(B)** Association plot centered on signal 2, using the lead SNP rs12513261as the index variant. Each plot displays SNPs within the GUCY1A1/GUCY1B1 locus on chromosome 4, colored by their linkage disequilibrium (LD, measured as *r* ^2^) with the respective lead SNP, based on the East Asian reference panel from the 1000 Genomes Project. SNPs in strong LD (*r* ^2^ ≥ 0.8) are shown in red, moderate LD (0.6 ≤ *r* ^2^ < 0.8) in orange, weak LD (0.4 ≤ *r* ^2^ < 0.6) in green, and very low LD (*r* ^2^ < 0.4) in light blue. Variants lacking LD information are shown in grey. Gene annotations and local recombination rates (blue curves) are provided for genomic context. Although both signals map to the same genomic region, their distinct LD structures suggest the presence of multiple independent regulatory elements at the GUCY1A1/GUCY1B1 locus.

Moreover, the region encompassing *GUCY1A1* and *GUCY1B1*—encoding key subunits of soluble guanylate cyclase (sGC), the intracellular receptor for nitric oxide—emerged as a candidate with dual associations to both MI and blood pressure traits (Table 3). This pathway mediates cGMP synthesis, promoting vasodilation and vascular tone regulation. Regional association analyses revealed evidence of allelic heterogeneity, with at least two statistically independent association peaks, implying the presence of multiple functional variants or distinct regulatory elements within this locus (Figure 2). Prior studies have linked this region to blood pressure phenotypes, and the cumulative burden of risk alleles in this region has been associated with heightened susceptibility to stroke and coronary disease. Our eQTL investigation using GTEx data further supports the functional relevance of this locus, as risk alleles were associated with reduced expression of *GUCY1A1* and *GUCY1B1* in arterial tissues, potentially impairing NO-sGC signaling and contributing to vascular dysfunction (Supplementary Table S5). These findings underscore the mechanistic convergence between endothelial signaling, hemodynamic regulation, and ischemic cardiac events.

**TABLE 3:** New pleiotropic loci identified in multi-trait GWAS of myocardial infarction.

SNP	CHR	BP	A1	A2	pval1	beta1	pval2	beta2	mtag_beta	mtag_pval	Gene	Trait 1	Trait 2
rs10029651	4	155510873	T	C	3.96E-07	−0.07	2.40E-04	−0.02	−0.02	3.43E-09	*GUCY1A1/GUCY1B1*	Myocardial infarction	Diastolic blood pressure
rs12646335	4	155501360	A	G	1.72E-06	−0.06	6.92E-05	−0.02	−0.02	8.79E-09	*GUCY1A1/GUCY1B1*		Systolic blood pressure
rs12513261	4	155692190	G	T	6.12E-07	0.07	9.33E-03	0.01	0.02	1.65E-08	*GUCY1A1/GUCY1B1*		
rs117123860	5	75299307	C	T	1.80E-07	0.17	4.37E-03	−0.03	0.04	1.58E-08	*HMGCR*		High density lipoprotein cholesterol measurement
rs1042085	12	10722926	A	G	5.63E-08	0.08	1.91E-03	−0.01	0.02	3.82E-09	*YBX3*		
rs3809129	12	56317587	T	C	1.63E-07	0.08	6.46E-03	−0.01	0.02	1.49E-08	*PAN2*		
rs76416614	12	113294519	A	G	7.07E-08	−0.09	8.32E-03	0.02	−0.02	6.08E-09	*TPCN1*		
rs11235672	11	73184057	C	T	1.13E-07	−0.13	6.76E-03	−0.02	−0.03	3.12E-09	*P2RY2*		Low density lipoprotein cholesterol measurement
rs7133315	12	109247311	G	A	2.86E-06	0.07	3.80E-06	0.02	0.02	8.82E-09	*ACACB*		Triglyceride measurement

SNP, Reference SNP cluster ID from dbSNP database; CHR, Chromosome location (hg38 assembly); BP, Base pair position (hg38 coordinates); A1, Effect allele (tested in association models); A2, Reference allele; pval1, Single-trait GWAS p-value for Trait 1; beta1, Effect size of A1 allele on Trait 1; pval2, Single-trait GWAS p-value for Trait 2; beta2, Effect size of A1 allele on Trait 2; mtag_beta, Multi-trait meta-analysis effect estimate; mtag_pval, Multi-trait analysis p-value; gene, Nearest protein-coding gene.

## 4 Discussion

In this study, we systematically dissected the shared genetic architecture of CVDs and cardiometabolic traits in East Asian populations through a multi-layered analytical framework. By integrating genome-wide genetic correlation analyses, local heritability mapping, tissue-specific enrichment profiling, and multi-trait genome-wide association studies, we identified several pleiotropic loci that illuminate converging biological mechanisms underlying cardiovascular and metabolic risk.

Our genetic correlation results affirm extensive shared heritability between key cardiovascular conditions. The strong correlations between HF and both systolic and diastolic blood pressure, as well as the consistent links between MI and LDL, TG, and HDL, support the notion that vascular dysfunction and lipid dysregulation are foundational to CVD pathogenesis. In contrast, the weaker correlations between arrhythmia and lipid traits suggest partially distinct genetic pathways for electrophysiological disorders. Our analysis also revealed extensive genetic connectivity among four major CVD phenotypes suggesting that these conditions, though clinically distinct, are influenced by overlapping genetic factors in East Asians. Notably, HF exhibited the strongest and most consistent genetic correlations with MI, AF and arrhythmia, underscoring its central role at the intersection of structural, electrical, and ischemic heart disease (Winata et al., 2025). These findings reflect clinical observations that HF often co-occurs with rhythm disturbances and evolves downstream of MI, serving as both a consequence and an amplifier of other cardiovascular pathologies. While MI showed moderate correlation with AF and strong correlation with HF, arrhythmia and AF were nearly genetically indistinguishable (rg = 1.0), consistent with their shared electrophysiological basis. Taken together, these results support a model in which HF acts as a genetically and pathophysiologically integrative phenotype, bridging hemodynamic overload, myocardial injury, and conduction abnormalities.

Critically, our MTAG analysis yielded several genome-wide significant pleiotropic loci, including established cardiovascular genes and novel signals with potential functional relevance. *APOB* encodes apolipoprotein B, the primary structural protein of LDL, which plays a central role in lipid transport and the formation of atherosclerotic plaques. Variants in *APOB* have been repeatedly linked to altered LDL-C levels and coronary artery disease risk across ancestries. In our analysis, rs13414987 at the *APOB* locus was jointly associated with both HF and MI, highlighting the gene’s pleiotropic effect on vascular integrity and downstream cardiac remodeling. This finding reinforces the contribution of lipid dysregulation to both atherosclerotic burden and cardiac decompensation, and suggests that *APOB*-mediated pathways may serve as early upstream determinants of adverse cardiovascular outcomes (Giannakopoulou et al., 2025; Vorster et al., 2025). Similarly, *MC4R* (melanocortin 4 receptor), a G protein–coupled receptor expressed predominantly in the hypothalamus, is known for its key role in regulating appetite and energy homeostasis. Common and rare variants in *MC4R* have been implicated in obesity, metabolic syndrome, and type 2 diabetes. In our study, the *MC4R* locus was significantly associated with both HF and MI, suggesting a broader influence of central energy regulation on cardiovascular risk. This observation supports the concept that neuroendocrine control of body weight and energy balance exerts long-term effects on cardiac structure and function, potentially through mechanisms involving insulin resistance, adiposity-driven inflammation, and neurohumoral activation (Guo et al., 2025). Notably, *MC4R* has also been linked to blood pressure regulation, further implicating this locus as a point of intersection between metabolic and hemodynamic stressors.

Beyond these well-characterized genes, we also identified novel pleiotropic signals with functional potential. Among them, *QSOX2* emerged as a previously unrecognized locus significantly associated with both HF and SBP. eQTL data revealed that rs3824359 modulates *QSOX2* expression in multiple tissues including arteries and the heart, suggesting a role for oxidative protein folding and redox balance in vascular homeostasis and cardiac stress response (Ning et al., 2024). Similarly, we identified distinct association peaks at the *GUCY1A1*/*GUCY1B1* locus, implicating reduced expression of nitric oxide receptor components in arterial tissues as a potential shared mechanism linking MI and elevated blood pressure (Vishnolia et al., 2021). These findings extend prior evidence on NO–sGC–cGMP signaling and offer mechanistic insights into the vascular origins of ischemic and hypertensive disease (Russo et al., 2024; Gawrys et al., 2025).

Our study has several limitations that should be acknowledged. First, although we identified multiple novel pleiotropic loci in East Asian populations, we were unable to perform replication in independent cohorts due to the limited availability of comparable datasets with matched phenotypes. Second, the GWAS summary statistics used in our analyses were derived from different studies, which may vary in terms of case definitions, diagnostic criteria, and phenotype ascertainment. Such heterogeneity in phenotype definitions could introduce noise and reduce the precision of association signals. Third, while some of the identified loci have been reported in European or other populations, many appear to be specific or stronger in East Asians. These differences may reflect ancestry-specific allele frequencies or linkage disequilibrium patterns, limiting the generalizability of our findings. Future studies involving harmonized phenotyping and multi-ancestry analyses will be critical to validate these associations and assess their relevance across diverse populations.

In conclusion, our study provides a comprehensive analysis of the genetic relationships among major CVDs and their cardiometabolic risk factors in East Asian populations. The identification of shared loci and enriched biological pathways emphasizes the interconnected nature of cardiovascular and metabolic disorders and highlights the need for integrative risk prediction and therapeutic strategies. Future efforts incorporating multi-omic data and trans-ancestry replication will be crucial to further refine these insights and translate them into precision medicine applications.

## Data Availability

The original contributions presented in the study are included in the article/Supplementary Material, further inquiries can be directed to the corresponding authors.
