# Supplementary material for: Shared genetic loci connect cardiovascular disease with blood pressure and lipid traits in East Asian populations
Source: Front Genet. 2025 Jun 24;16:1635378. doi: 10.3389/fgene.2025.1635378 (PMC12234571; doi:10.3389/fgene.2025.1635378)

Supplementary Figure 1. Heat maps showing patterns of cell-type-specific enrichments of SNP-heritability for genetically associated traits across 396 cell-type-specific annotations. Each checkered rectangle reflects the z-score, scaled by traits. Red indicates enrichment, blue indicates depletion. Deeper color represents stronger magnitude of effects. Asterisks represent statistical significance withstanding multiple correction. The category of cell-types is color coded to the left. A) DNase (DNase I hypersensitive sites), B) H3K27ac, C) H3K36me3, D) H3K4me1, E) H3K4me3, and F) H3K9ac.

A) DNase


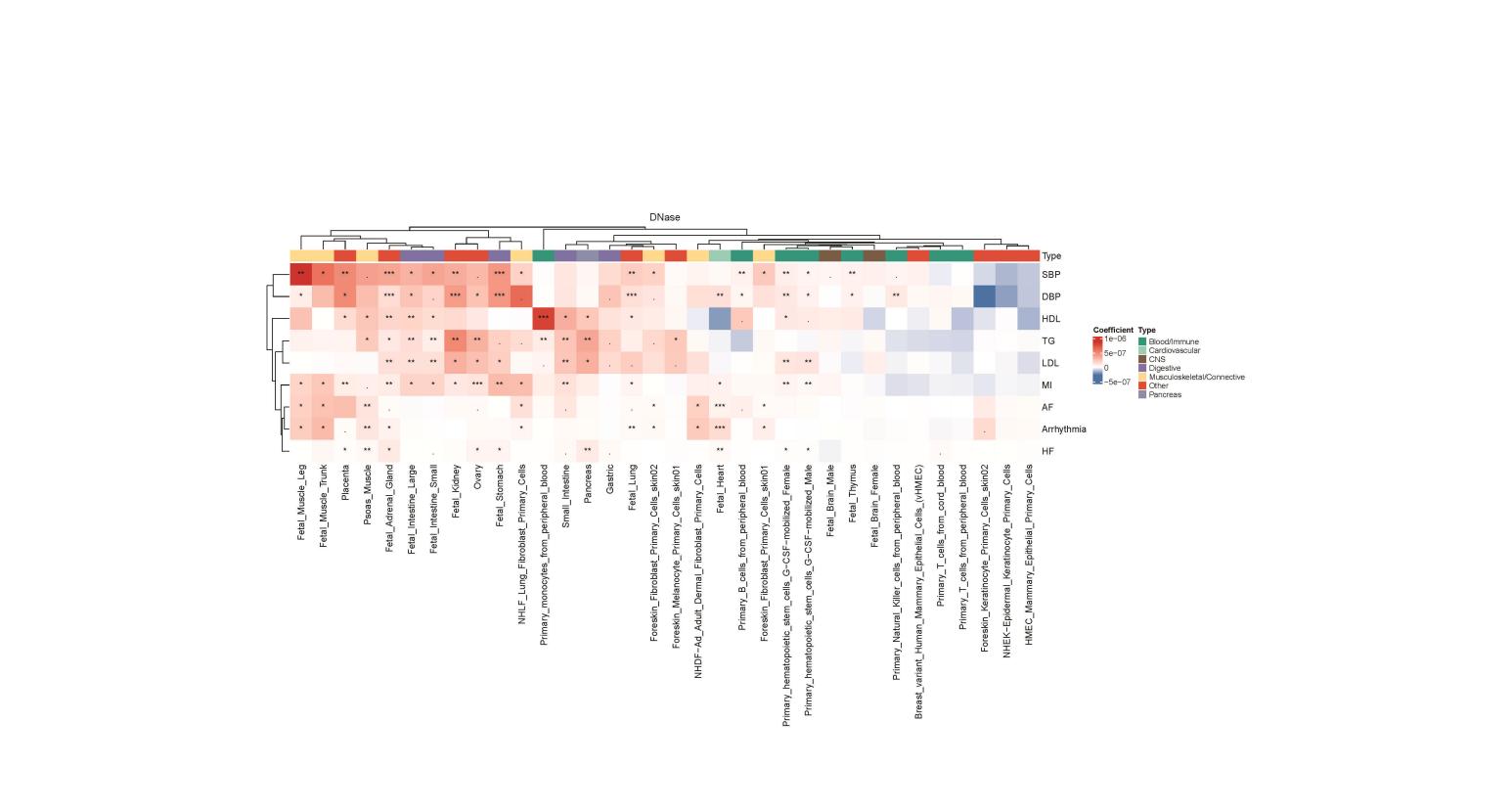


B) H3K27ac


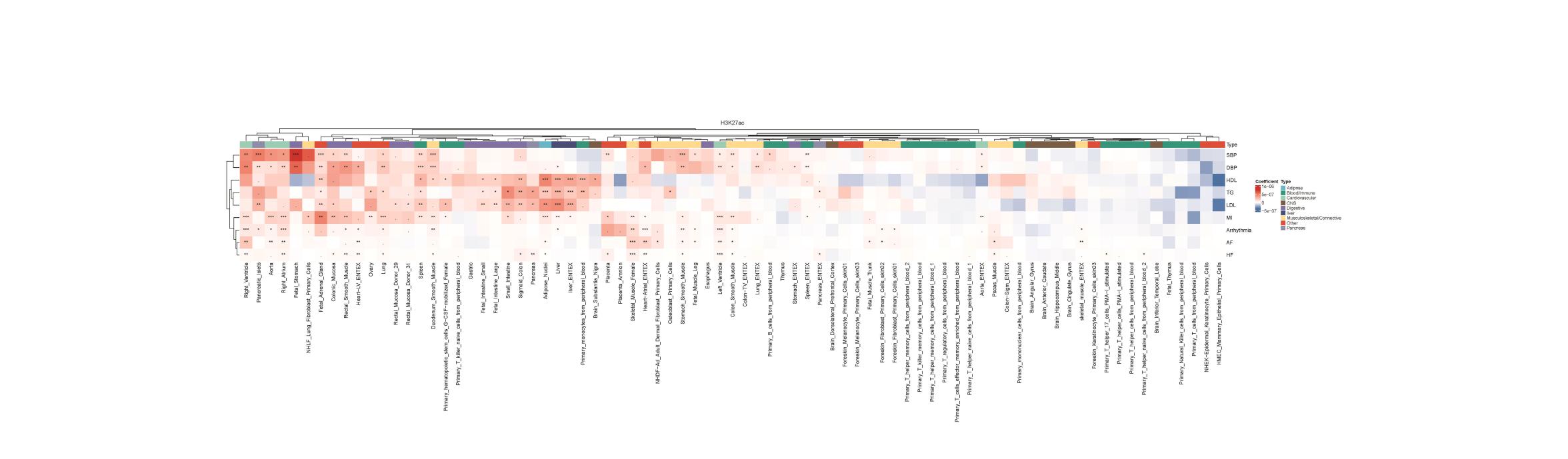


C) H3K36me3


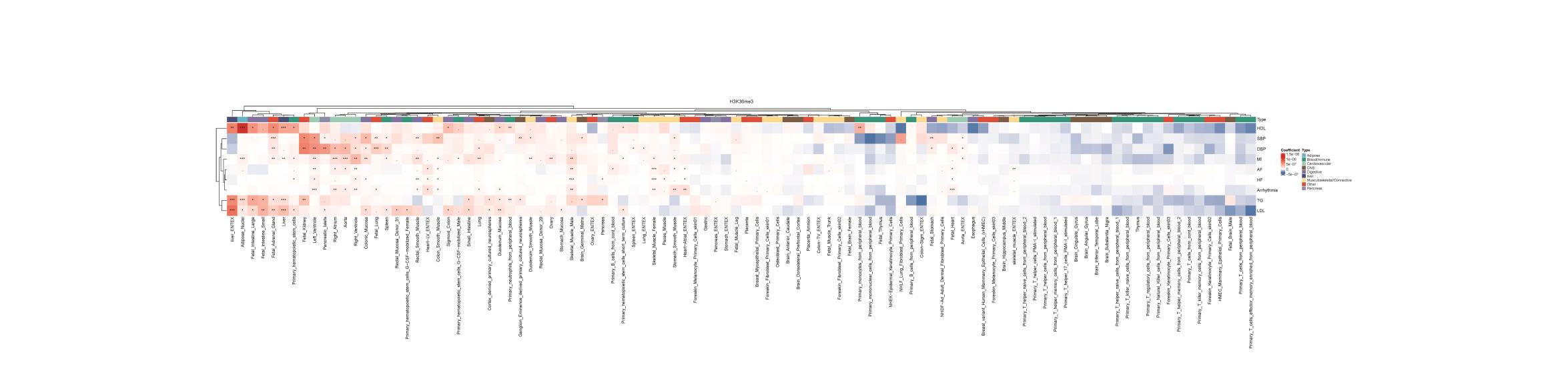


D) H3K4me1


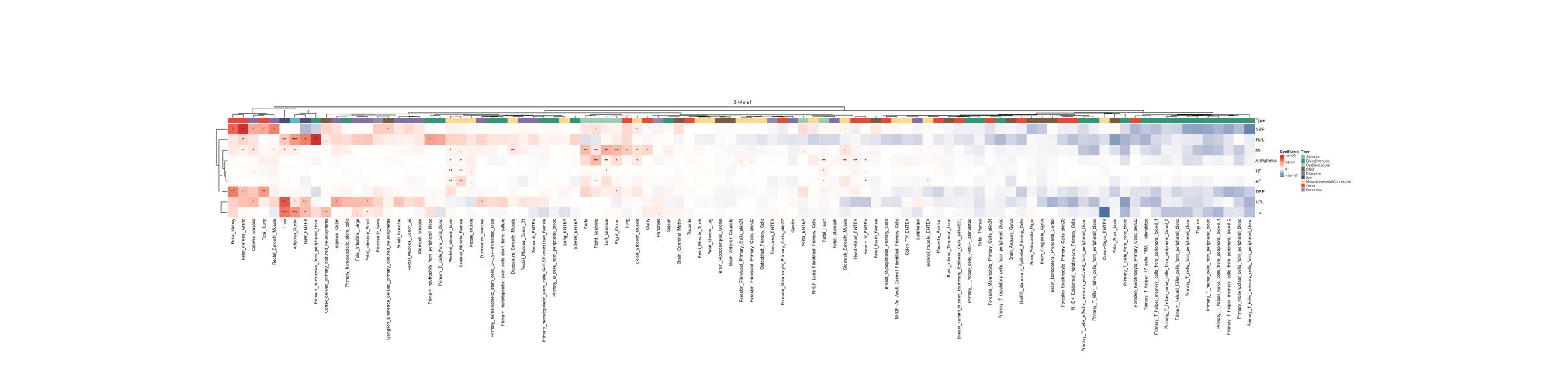


1. H3K4me3


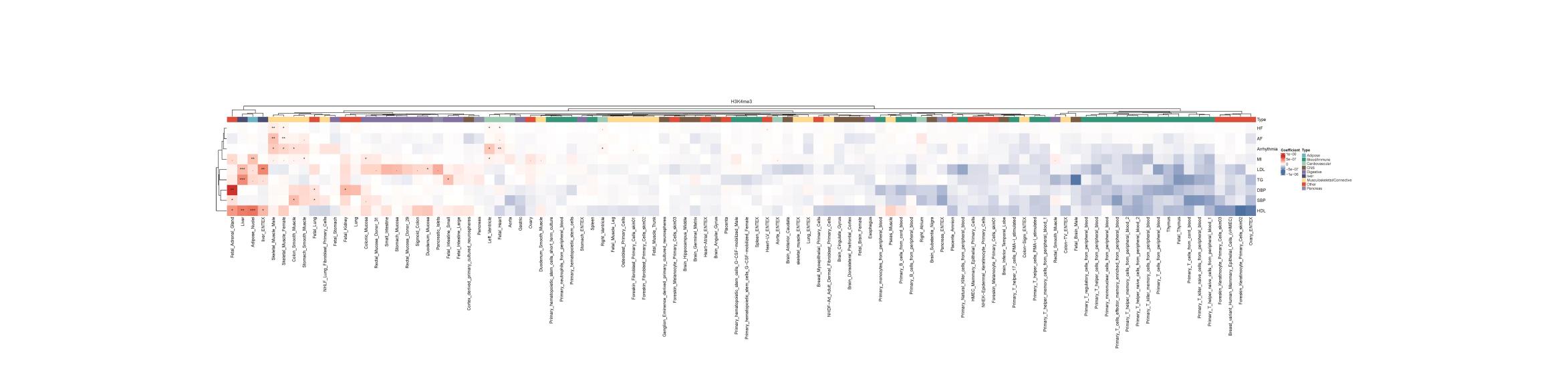


F) H3K9ac


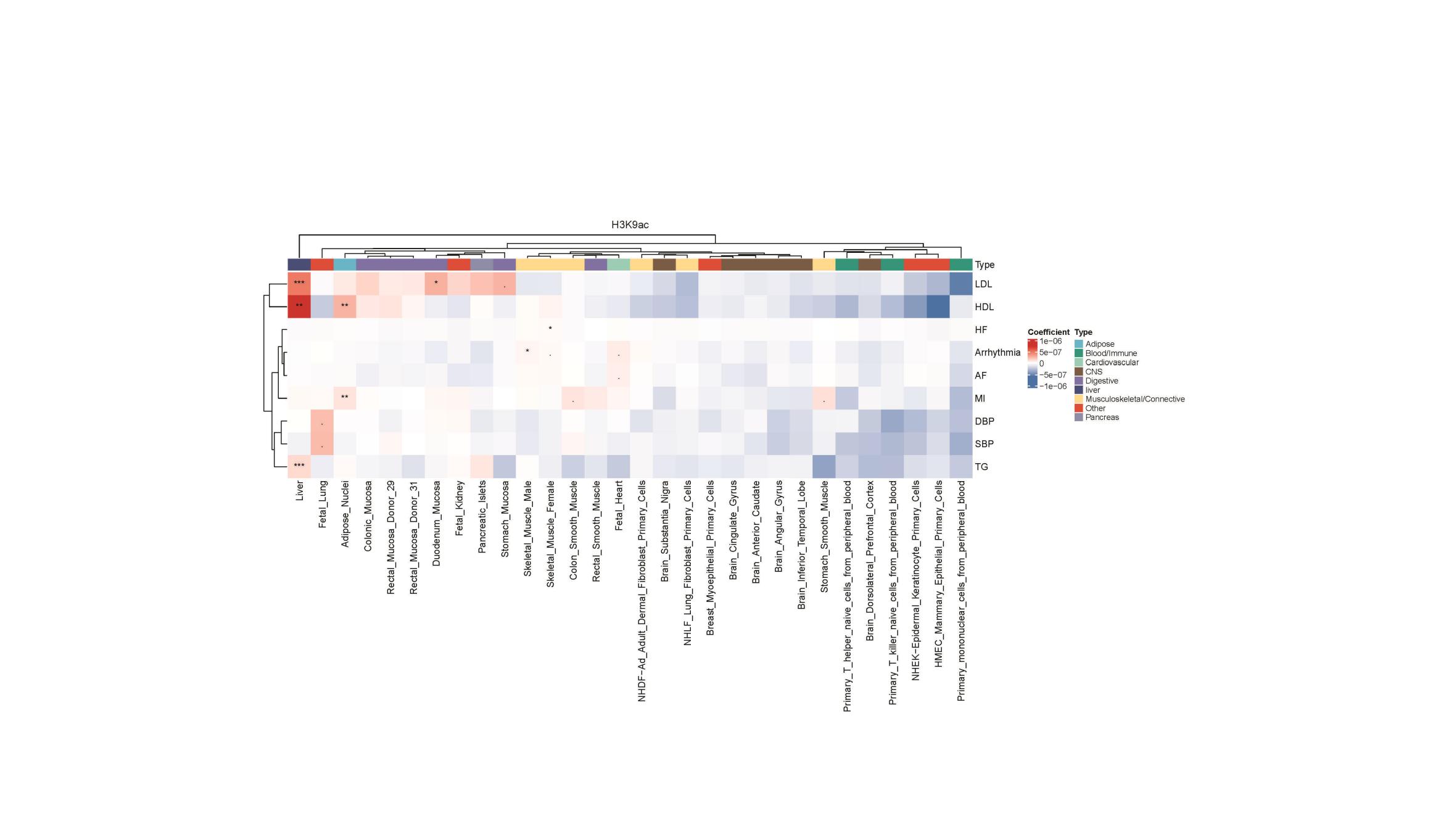


Supplementary Figure 2. Regional plots for the associations results. SNPs are colored according to their linkage disequilibrium (LD, measured as r²) with the respective lead variant, using the East Asian reference panel from the 1000 Genomes Project. Variants in strong LD (r² ≥ 0.8) are shown in red, those in moderate LD (0.6 ≤ r² < 0.8) in orange, weak LD (0.4 ≤ r² < 0.6) in green, and very low LD (r² < 0.4) in light blue. SNPs lacking LD data are shown in grey. Local recombination rates (blue curves) and gene tracks are displayed to provide genomic context.

A)


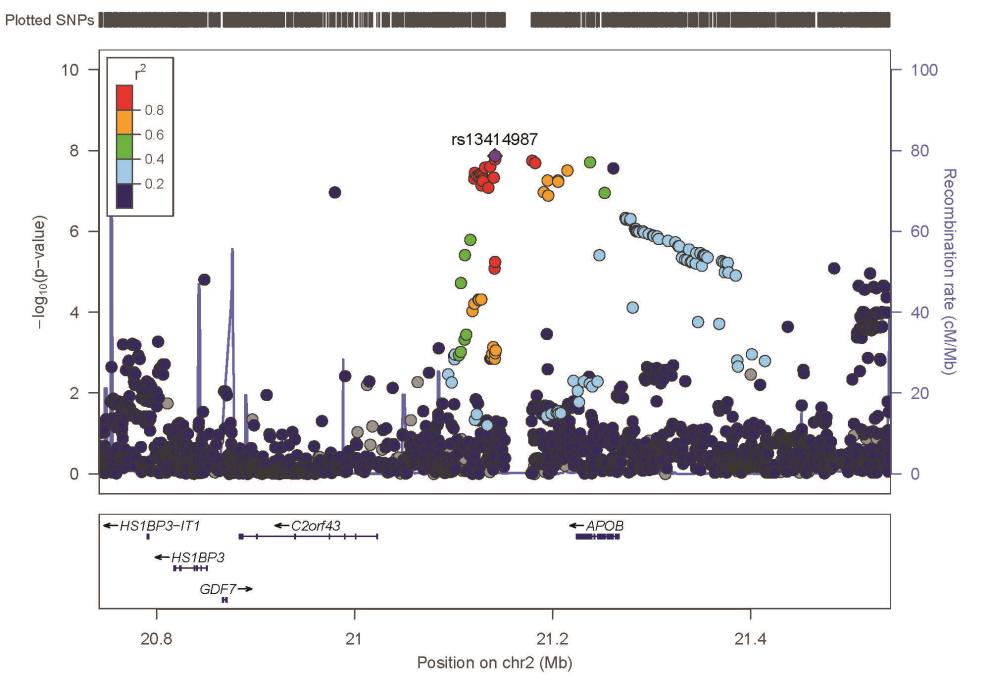


B)


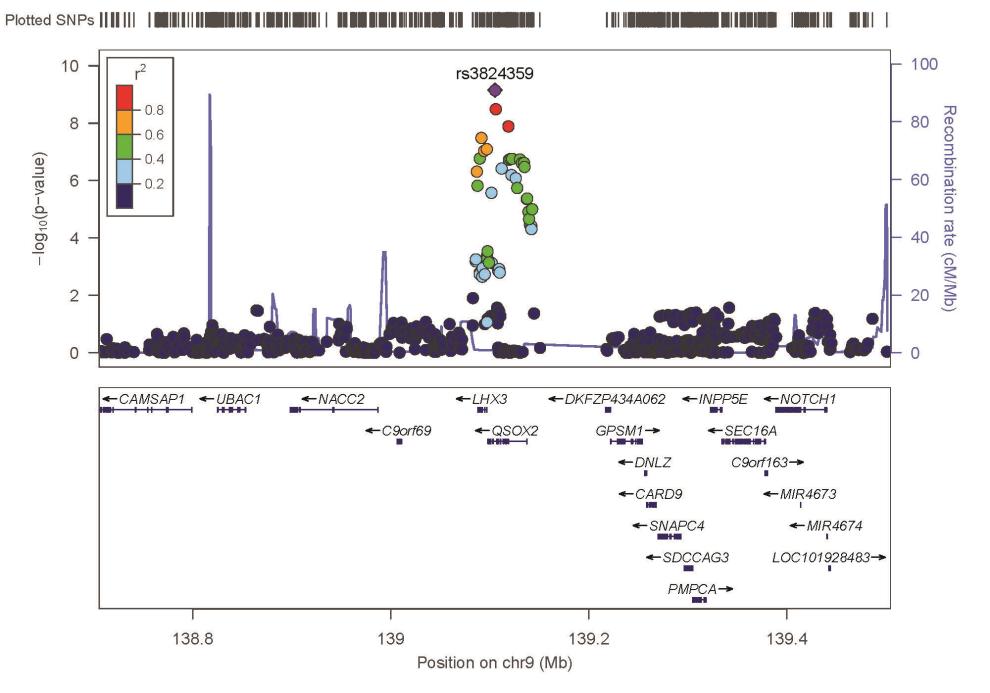


C)


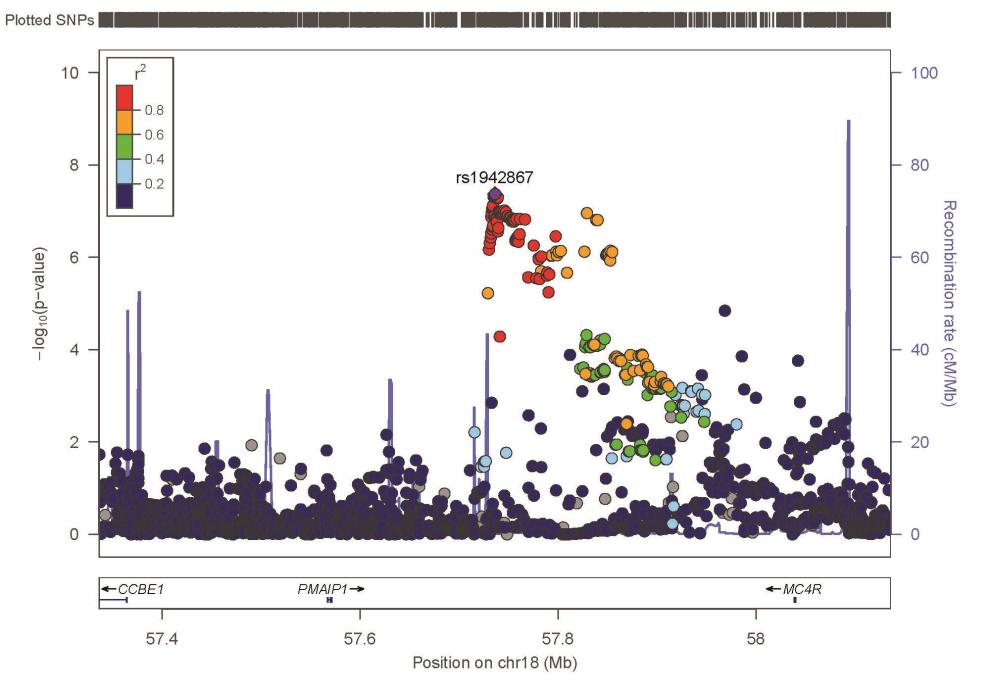

Supplement: Supplementary file 1 [file Supplementaryfile1.docx]
